# A systematic review of penetrating injuries to the superior sagittal sinus across two centuries

**DOI:** 10.3389/fneur.2026.1789736

**Published:** 2026-06-11

**Authors:** Jarett E. Prince, Kivanc Yangi, Kashif Qureshi, Michell Goyal, Jack T. Olson, Egemen Gok, Mark C. Preul

**Affiliations:** The Loyal and Edith Davis Neurosurgical Research Laboratory, Barrow Neurological Institute, St. Joseph’s Hospital and Medical Center, Phoenix, AZ, United States

**Keywords:** dural venous sinus, historical, penetrating brain injury, sinus injury management, superior sagittal sinus, surgical techniques, systematic review, venous sinus repair

## Abstract

**Objective:**

Penetrating injuries of the superior sagittal sinus (pSSSIs) are rare but catastrophic, often causing massive hemorrhage, intracranial hypertension, and neurological decline. Despite centuries of reported cases, management guidance remains fragmented. This study reports a systematic literature review that sought to characterize pSSSIs by mechanism, anatomical involvement, surgical management, and outcomes and trace the evolution of repair techniques over 2 centuries.

**Methods:**

PubMed, Embase, Scopus, and Cochrane databases were searched in accordance with Preferred Reporting Items for Systematic Reviews and Meta-Analyses guidelines. Inclusion criteria targeted original reports of pSSSIs, excluding iatrogenic or nonpenetrating trauma. Study quality was assessed using the Joanna Briggs Institute tools.

**Results:**

Thirty-nine articles describing 51 cases for the period 1826–2025 were included. Patients were predominantly male (49 of 51; 96%) with a mean (SD) age of 30.3 (15.1) years. Causes included military trauma (33%), accidents (29%), suicides (18%), and assaults (8%). The middle third of the sinus was most often affected (67%), followed by the anterior (24%) and posterior (18%) thirds. Nails (24%), bone fragments (16%), and bullets (16%) were frequent penetrating objects. Repair methods included hemostatic agents (25%), grafts (25%), ligation (14%), and sutures (14%). Mortality was 27% and was highest among individuals with injuries to the anterior third of the sinus and those with complex trauma.

**Conclusion:**

pSSSIs are uncommon but life-threatening, with outcomes determined by the anatomical site, mechanism, and timely intervention. Although surgical management options have evolved, no standardized paradigm exists. Contemporary approaches emphasize tailored combinations of direct repair, reconstruction, and selective ligation. Further work is needed to establish consensus guidelines and optimize outcomes in these challenging cases.

**Systematic review registration:**

https://www.crd.york.ac.uk/PROSPERO/view/CRD420251124767, identifier (CRD420251124767).

## 1 Introduction

The superior sagittal sinus (SSS) is one of the earliest anatomical landmarks to be described, first documented by Herophilus of Chalcedon (335–280 BC) (1, 2). In the 1930s, Herbert Olivecrona (1891–1980) and Harvey Cushing (1869–1939) divided the sinus into anterior, middle, and posterior thirds, each with distinct vascular segmentation and collateral drainage (1, 3–5). This division is not only of historical interest but also of clinical relevance, because injuries to different thirds of the SSS present unique surgical challenges.

The SSS is most often injured in traumatic brain injury (TBI), where it accounts for 70–80% of dural venous sinus injuries (6). Laceration and compression from hematomas or displaced bone fragments can lead to hemorrhage, thrombosis, intracranial hypertension, and neurological decline (1, 6–8). Penetrating TBIs (pTBIs), in which an object breaches the dura and brain parenchyma (9–11), or in which the sinus itself is traversed, are less common than blunt TBIs but carry poor outcomes, with mortality up to 42% (12–15). Penetrating SSS injuries (pSSSIs) represent a particularly severe subset of these injuries.

Given this high mortality, attempts to refine and perform the repair of these injuries have been documented by surgeons for nearly 2 centuries (16). Approaches include direct repair with sutures or clips, autologous grafts or flaps, and sinus ligation (17, 18). However, a standardized framework remains absent, and treatment is still guided by individual case factors. This study addresses that gap by synthesizing reported cases to define injury patterns, management strategies, and outcomes across historical and modern practice.

## 2 Materials and methods

This study adhered to the Preferred Reporting Items for Systematic Reviews and Meta-Analyses (PRISMA) (19) guidelines and was registered in the PROSPERO international prospective register of systematic reviews (registration ID: CRD420251124767).

### 2.1 Search strategy

Systematic searches of PubMed, Embase, Scopus, and the Cochrane Library were conducted on August 27, 2025, using the following query, with no time restrictions applied: (((((((Sagittal Sinus, Superior) OR (Sinus, Superior Sagittal)) OR (Sinus Sagittalis Superior)) OR (Superior Longitudinal Sinus)) OR (Longitudinal Sinus, Superior)) OR (Sinus, Superior Longitudinal)) OR (Superior Sagittal Sinus)) AND (((((Penetrant) OR (Penetrating)) OR (Penetrant Injury)) OR (Penetrating Injury)) OR (Injury, Penetrating)). These medical subject heading terms were combined using the Boolean operators “AND” and “OR” to construct a comprehensive search strategy. Adjustments were applied to the search strategy to comply with the advanced search parameters of the respective databases.

### 2.2 Eligibility criteria

The inclusion criteria were based on original research articles detailing pSSSIs. In accordance with classifications in the literature (9–11), pSSSIs were defined as injuries in which an object, such as a projectile, sharp implement, or bone fragment, penetrates the sinus. Only publications with a clearly described mechanism of injury and definitive involvement of the SSS were included. We elected to exclude articles that focused on other forms of injury, pTBIs without SSS involvement, pSSSIs of an iatrogenic nature, or cases in which the mechanism of injury or extent of sinus involvement were unclear or insufficiently described (e.g., abutting or adjacent injuries without confirmed penetration). We also excluded articles with cohorts that lacked a clear delineation of patient data as well as review articles, commentaries, editorials, retracted publications, and errata.

### 2.3 Study selection

Upon retrieving the results of our query, duplicates were excluded, and all remaining articles were screened. Both the reference-check method and a manual search were used to identify additional articles that met our inclusion criteria. All articles were uploaded to the Rayyan platform (20) and screened by 2 independent reviewers (MG, JTO). After screening, any disagreements were resolved by a third reviewer (KY), and 39 articles were deemed eligible for inclusion.

### 2.4 Data extraction

Data extraction from each included article was performed by 2 authors (JEP, KY). Retrieved data included the case year, patient demographics, type of penetrating object, segment of the SSS involved, admission neurological status, techniques for SSS bleeding control and repair, neurological outcome, length of hospital stay, follow-up duration, and mortality. The involved SSS segment data were either collected as stated by the study authors or recorded based on the reviewers’ analysis of radiographs and case descriptions. Admission neurological status was recorded as a Glasgow Coma Scale (GCS) score when included by the study authors. For earlier texts that relied on narrative description, the reviewers recorded a comprehensive summary. For each outcome domain, all results reported in each study were extracted when available. For neurological outcomes, we recorded the discharge status as the primary time point for collection. Length of hospital stay and follow-up duration were extracted as stated by the study authors. However, when multiple time points were available, the most comprehensive value was extracted. For mortality, both in-hospital and follow-up deaths relating to the pSSSI or sequelae were recorded. All other variables were collected as written by the study authors. The collected data were arranged in Table 1 (6, 16, 18, 21–56).

**Table 1:** Summary of studies focused on penetrating injuries of the superior sagittal sinus.

Study	Case year	Pt age (y), sex	Penetrating object	Segment of SSS involved	Admission neurological status	SSS bleeding control and repair techniques	Neurological outcome	LOS	F/u duration	Survived
Toogood (16)	1826	44 M	Bone fragment	Anterior	Incoherent	Packing material (lint)	No deficits	NS	NS	Y
Cole (21)	1848	19 M	Tree branch	Middle and posterior	Somnolent; incoherent; pupils dilated but reactive	Packing material (muslin)	No deficits	NS	NS	Y
Hopkins (22)	1884	32 M	Bone fragment	Middle	Unconscious	Packing material (lint), suture (unsuccessful)	Memory loss	71 d	NS	Y
Rawdon (23)	1892	17 F	Iron spike	NS	Unconscious	Suture (catgut)	Visual impairment; pupils are dilated	114 d	NS	Y
Keen (24)	1893	NS M	Bone fragment	Middle and posterior	No deficits	Instrument-based hemostasis (forceps), packing material (iodoform gauze)	Left homonomous hemianopsia	NS	2.5 y	Y
Holmes and Sargent (25)	1914	NS M	Bullet	Middle	Dull; apathetic; LSS	NS	Weakness and ataxia in both arms; hyperreflexia; multiple sensory deficits; LSS	5 w	NS	Y
Cushing (26)	1917	NS M	NS	Anterior	Complete loss of inhibition	NS	Deceased	7 d	NA	N
Cushing (26)	1917	NS M	Bone fragment	Anterior	Incoherent; hyperactive reflexes	Instrument-based hemostasis (silver clip)	No deficits	31 d	73 d	Y
Cushing (26)	1917	NS M	Shrapnel	Middle	Left hemianopsia, spastic hemiplegia, and hemihypoesthesia; facial weakness	Graft (autologous)	Deceased	84 d	NA	N
Cushing (26)	1917	NS M	NS	Middle	Spastic paraplegia	NA	Deceased	NA	NA	N
Cushing (26)	1917	NS M	Shrapnel	Middle	Spastic paresis of all 4 extremities (more marked in LLE) with sensory deficits; LSS	Instrument-based hemostasis (silver clip)	Mild LLE weakness	>129 d	NS	Y
Cushing (26)	1917	NS M	Shrapnel	Middle	Unconscious; LSS	NS	Deceased	1 d	NA	N
Cushing (26)	1917	NS M	Shrapnel	Posterior	Right hemiparesis	Graft (autologous)	Deceased	10 d	NA	N
Horrax (27)	1918	NS M	Bone fragment	Anterior	Disoriented; frontal lobe syndrome	Graft (autologous)	No deficits	22 d	36 d	N
Horrax (27)	1918	NS M	Bullet	Anterior	Semiconscious; disoriented; left hemiplegia; DTR hyperactive on left; ankle and jaw-clonus	Graft (autologous)	Deceased	43 d	NA	N
Horrax (27)	1918	NS M	Bone fragment	Middle	Right hemiplegia and hemihypoesthesia; drowsy; irrational with aphasia	Graft (autologous)	Deceased	11 d	NA	N
Horrax (27)	1918	NS M	Bone fragment	Middle	Right hemiplegia; DTR hyperactive bilaterally; Babinski positive on right	NS	Deceased	11 d	NA	N
Horrax (27)	1918	NS M	Bullet	Middle	Unconscious	NS	Deceased	20 d	NA	N
Horrax (27)	1918	NS M	Bullet	NS	Left paraparesis	Graft (autologous)	Mild weakness	116 d	NS	Y
Kapp et al. (28)	1971	39 M	Shrapnel	Posterior	Cortical blindness; left hemiplegia; DTR hyperactive bilaterally; Babinski positive bilaterally	Graft (autologous), dural venous shunt, suture	Left brachial plegia	NS	NS	Y
Brisman and Harrington (29)	1971	30 M	Shrapnel	Posterior	Stuporous; right hemiparesis; left Bell’s palsy	Graft (autologous)	Ophthalmoplegia (“1.5” syndrome); ataxic; unsteady gait	15 d	NS	Y
Olumide and Adeloye (30)	1974	24 M	Nail	Middle	Paraparesis	Hemostatic agent (Surgicel)	No deficits	10 d	Lost to F/u	Y
Haßler (56)	1979	NS F	Bullet	Anterior	NS	NS	Deceased	NS	6 mo	N
Nehme (31)	1974	15 M	Bullet	Middle	Comatose; anisocoria (right pupil fixed and dilated)	NS	No deficits	26 d	1 y	Y
Nagahiro et al. (32)	1981	47 M	Nail	Middle	No deficits	Hemostatic agent (Oxycel)	Mild hypoesthesia and paresthesia of left foot	NS	NS	Y
Wu and Shih (33)	1979	27 M	Nail	Middle	No deficits	Hemostatic agent (Gelfoam)	No deficits	NS	14 mo	Y
Wu and Shih (33)	1979	42 M	Nail	Middle	Stuporous and mild right hemiparesis (more prominent on leg)	Hemostatic agent (Gelfoam), Suture	No deficits	NS	12 mo	Y
Sani et al. (34)	2005	37 M	Nail	Middle	Anomic aphasia	Graft (autologous)	No deficits	3 d	6 mo	Y
Judd and Wyatt (35)	2007	47 M	Saw blade	NS	Deceased	NA	Deceased	NA	NA	N
Balak et al. (36)	2009	9 M	Marble fragment	Middle	GCS 13; right hemiparesis	Hemostatic agent (Surgicel)	Right hemiparesis	7 d	NS	Y
Mathew and Sharma (37)	2010	7 M	Tile fragment	Anterior	GCS 15	Hemostatic agent (Gelfoam, Surgicel)	No deficits	NS	Lost to F/u	Y
Sedney et al. (38)	2012	4 M	Nail	Middle	No deficits	Ligation	No deficits	NS	5 mo	Y
Fischer et al. (39)	2012	19 M	Knife	Middle	No deficits	Graft (autologous)	No deficits	7 d	NS	Y
Khursheed et al. (40)	2013	20 M	Bone fragment	Anterior and middle	GCS 13	Hemostatic agent (Gelatin)	GCS 15	21 d	8 mo	Y
Hoffmann et al. (41)	2014	20 M	Bullet	Middle	GCS 3	Ligation	Cognitive deficits	3 mo	NS	Y
Kim et al. (42)	2015	23 M	Iron pipe	Middle	GCS 14	NS	Deceased	NA	NA	N
Ramos et al. (43)	2017	55 M	Drill bit	Middle and posterior	GCS 9	Ligation	GCS 15	NS	2 mo	Y
Sheng et al. (6)	2017	22 M	Knife	Posterior	GCS 15	Conservative	No deficits	7 d	9 mo	Y
Guppy and Ochi (44)	2018	30 M	Screw	Middle	GCS 13	NS	No deficits	3 d	3 y	Y
Brune et al. (45)	2018	39 M	Bullet	Middle	GCS 3	Conservative	GCS 3	9 d	NA	N
Arham and Zaragita (46)	2021	3 M	Nail	Middle	No deficits	Dural flap, hemostatic agent, instrument-based hemostasis (clip)	No deficits	7 d	6 mo	Y
Zima et al. (47)	2022	55 M	Nail	Anterior	NS	Hemostatic agent (foam-based), packing material (cottonoid patties)	Right hemiparesis	NS	NS	Y
Abdallah et al. (48)	2022	33 M	Shrapnel	Middle and posterior	NS	Balloon tamponade, graft (synthetic), hemostatic agent (Gelfoam), instrument-based hemostasis (clip)	GCS 15	5 d	2 w	Y
Schlag et al. (49)	2022	56 M	Coin	Middle	GCS 9	Hemostatic agent (fibrin glue), ligation	No deficits	10 d	NS	Y
Kow et al. (50)	2023	35 M	Nail	Anterior	GCS 15	Ligation	No deficits	NS	8 w	Y
Nussbaum et al. (18)	2023	45 M	Nail	Middle	No deficits	Suture (Nurolon)	No deficits	NS	1 mo	Y
Somrani et al. (51)	2023	30 M	Rake tooth	Middle	GCS 14	Graft (autologous), instrument-based hemostasis (MacKenzie clip), packing material	RUE monoparesis	15 d	NS	Y
Fujiyama et al. (52)	2024	25 M	Nail	Middle	GCS 15	Graft (synthetic), hemostatic agent (fibrin glue)	No deficits	NS	5 mo	Y
Zhu et al. (53)	2025	24 M	Bolt gun rod	Anterior	GCS 13	Ligation	Mild cognitive deficit	24 d	5 mo	Y
Baig et al. (54)	2025	61 M	Knife	Anterior and middle	GCS 15	Ligation, suture (silk)	No deficits	NS	NS	Y
Ekpene et al. (55)	2025	27 M	Nail	Middle and posterior	GCS 10; right hemiparesis	Hemostatic agent (Surgicel), suture (tack-up)	No deficits	21 d	>1 y	Y

d, days; DTR, deep tendon reflexes; F, female; F/u, follow-up; GCS, Glasgow Coma Scale score; LLE, left lower extremity; LOS, length of hospital stay; LSS, Longitudinal Sinus Syndrome; M, male; mo, months; NA, not applicable; NS, not specified; Pt, patient; RUE, right upper extremity; SSS, superior sagittal sinus; w, weeks; y, years.

### 2.5 Quality assessment and grading of evidence

The Joanna Briggs Institute (JBI) Critical Appraisal Checklists were used to analyze the methodological quality and potential bias of our included studies. Thirty-five studies were evaluated using the JBI tool for case reports (57), and 4 studies were evaluated using the JBI tool for case series (58). These tools were selected for their structured approach to evaluating internal validity and relevance within their respective study designs. Two independent reviewers (JEP, KQ) assessed each question. A third reviewer (M. G.) addressed any remaining unresolved disputes until an agreement was found. We also evaluated the included studies using the American Association of Neurological Surgeons/Congress of Neurological Surgeons (AANS/CNS) evidence grading framework, categorizing each as class I, II, or III (59).

## 3 Results

### 3.1 Selection process

**Figure 1:**
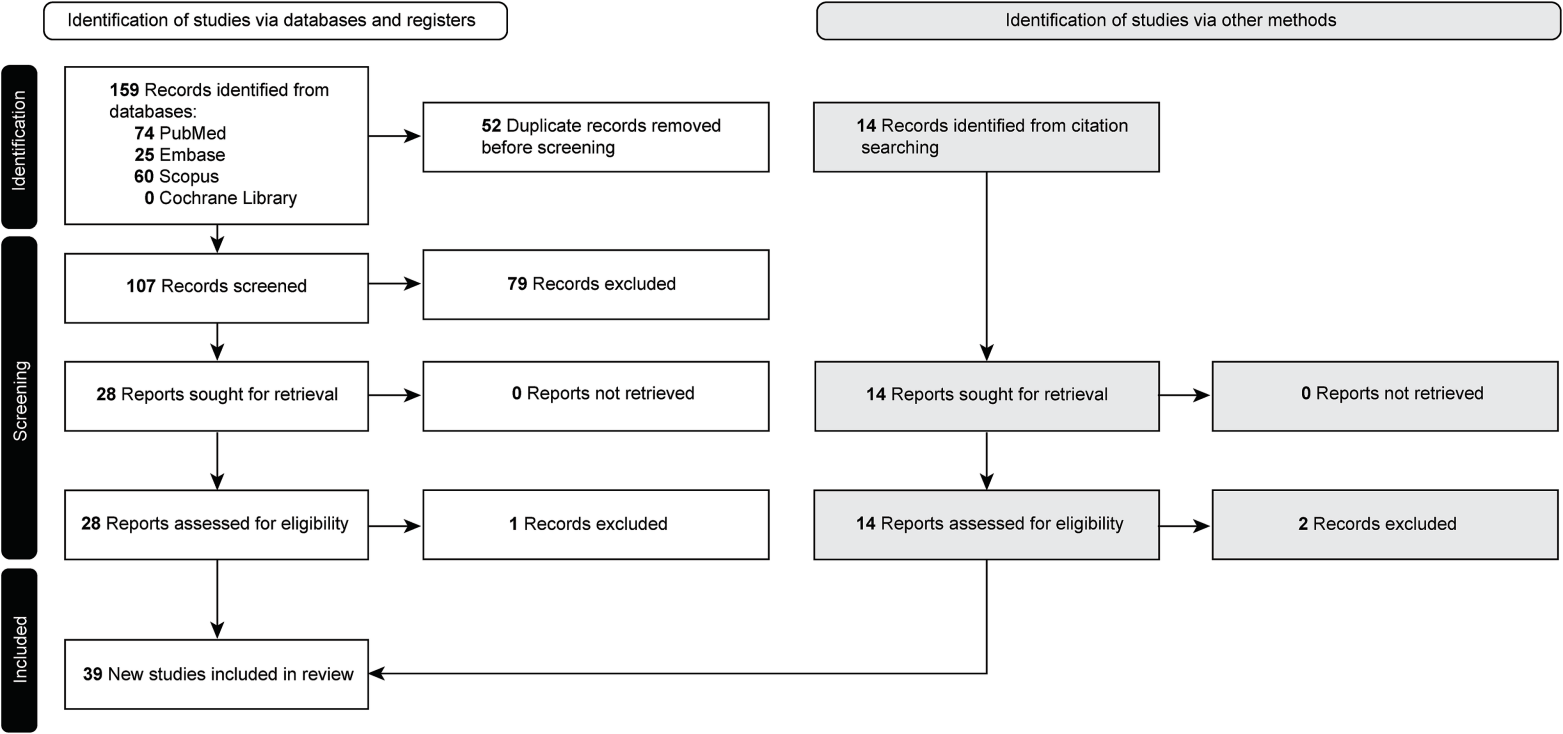
Flow diagram documenting the study selection process. Used with permission from Barrow Neurological Institute, Phoenix, Arizona.

A total of 159 records were identified through database searches, with 52 duplicates removed before screening. Of the 107 unique records, 79 were excluded after title and abstract review, leaving 28 articles for full-text assessment. One of these articles was excluded because it did not meet the eligibility criteria. An additional 14 studies were identified through citation searching, of which 2 were excluded for not meeting the inclusion criteria. Ultimately, 39 studies met the inclusion criteria and were incorporated into the review (Figure 1).

### 3.2 Quality assessment and grading of evidence

Of the 39 included studies, 35 were case reports, and 4 were case series (26, 27, 33). The majority (36 of 39; 92%) were rated as having a low risk of bias, and 3 case series were rated as having a moderate risk of bias (26, 33, 56). No study was classified as having a high risk of bias. Overall, the quality of evidence was acceptable for synthesis. A summary of the results for each assessment is presented in Supplementary Table 1 (6, 16, 18, 21–55). All included articles were categorized as class III evidence according to the AANS/CNS criteria, primarily due to their nature as case studies and case reports, which lack control groups and rely on descriptive clinical experience (Supplementary Table 2) (59).

### 3.3 Patient data

**Figure 2:**
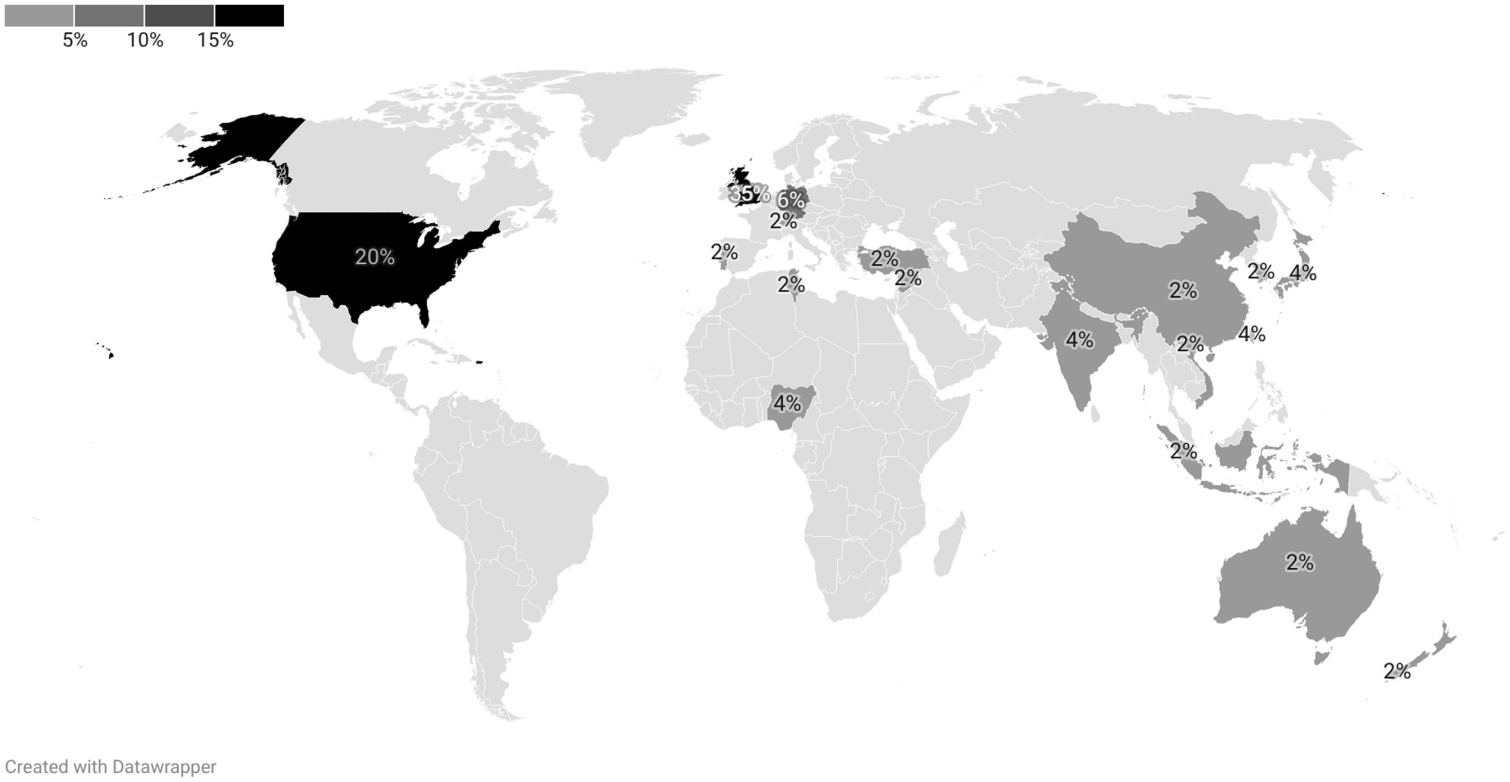
Case origins. A map representing the 19 countries from which the cases in our analysis were derived, highlighted according to their frequency of appearance. These include (in order of prevalence): United Kingdom (35%), United States (20%), Germany (6%), India (4%), Japan (4%), Nigeria (4%), Taiwan (4%), Afghanistan (2%), Australia (2%), China (2%), Indonesia (2%), New Zealand (2%), Portugal (2%), South Korea (2%), Switzerland (2%), Syria (2%), Tunisia (2%), Turkey (2%), and Vietnam (2%). Map created with Datawrapper (https://www.datawrapper.de).

Fifty-one cases of pSSSI were identified in the 39 included articles (Table 1), with reports published between 1846 and 2025 (6, 16, 18, 21–55). In 24 cases, the precise year of the operation was documented, providing a clearer chronological framework between 1826 and 2025 (16, 21–27, 29–31, 33). The included cases were derived from 19 countries, predominantly from the United Kingdom (18 of 51; 35%) and the United States (10 of 51; 20%) (Figure 2). Patient ages ranged from 3 to 61 years, with a mean (SD) age of 30.3 (15.1) years. Of 51 cases, only 4 were pediatric (8%), whereas the remaining 47 were adult (92%) (24–27). Forty-nine patients (96%) were male, and only 2 (4%) were female.

### 3.4 Injury and management

**Figure 3:**
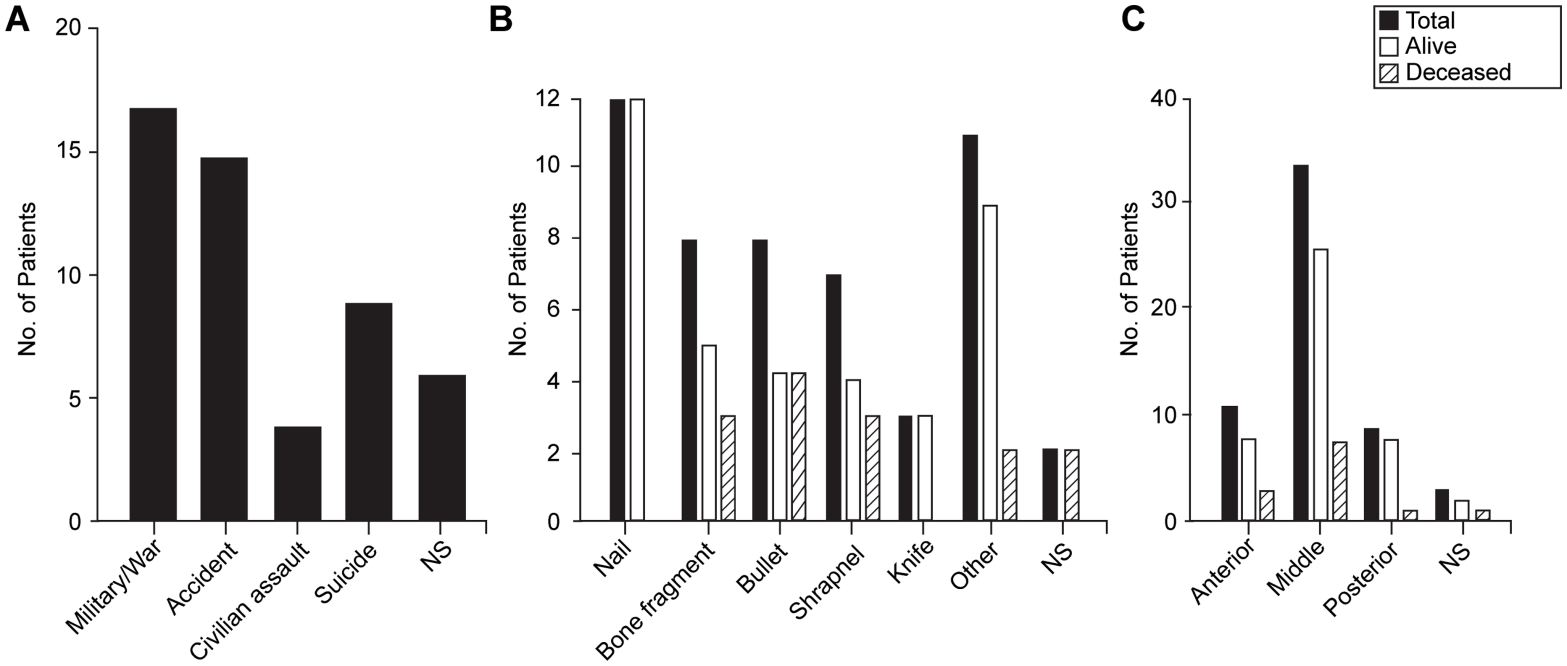
Bar charts illustrating trends in the etiology of penetrating injury of the superior sagittal sinus (pSSSI). **(A)** Mechanisms of injury in pSSSI. **(B)** Patient mortality with respect to the penetrating object. **(C)** Patient mortality with respect to the 3 segments of the superior sagittal sinus (SSS). Cases that presented with an injury to 2 segments of the SSS were included in both sections of the graph. NS, not specified. Used with permission from Barrow Neurological Institute, Phoenix, Arizona.

Causes for the pSSSIs included military or wartime trauma (*n* = 17; 33%), accidents (*n* = 15; 29%) (e.g., falls, construction accidents), suicide attempts (*n* = 9; 18%), and civilian assault (*n* = 4; 8%) (Figure 3A). In 6 cases, the cause of the injury was not specified (25, 34, 40, 43, 45, 56). The most common penetrating object was a nail (*n* = 12; 24%), followed by bone fragments (*n* = 8; 16%), bullets (*n* = 8; 16%), shrapnel (*n* = 7; 14%), and knives (*n* = 3; 6%), with 11 other objects (22%) uniquely featured (Figure 3B). In 2 cases (4%), the penetrating object was not clearly specified (26).

The majority of injuries involved the middle third of the SSS (*n* = 34; 67%), with fewer cases affecting anterior (*n* = 12; 24%) or posterior (*n* = 9; 18%) segments (Figure 3C). In 3 reports, segment involvement was not clearly specified (23, 27, 35), whereas 7 cases had 2 segments involved and were therefore included more than once in each respective segment within our analysis (21, 24, 40, 43, 48, 54, 55). Two patients had combined anterior and middle segment injuries (40, 54), whereas 5 had combined middle and posterior segments (21, 24, 43, 48, 55).

**Figure 4:**
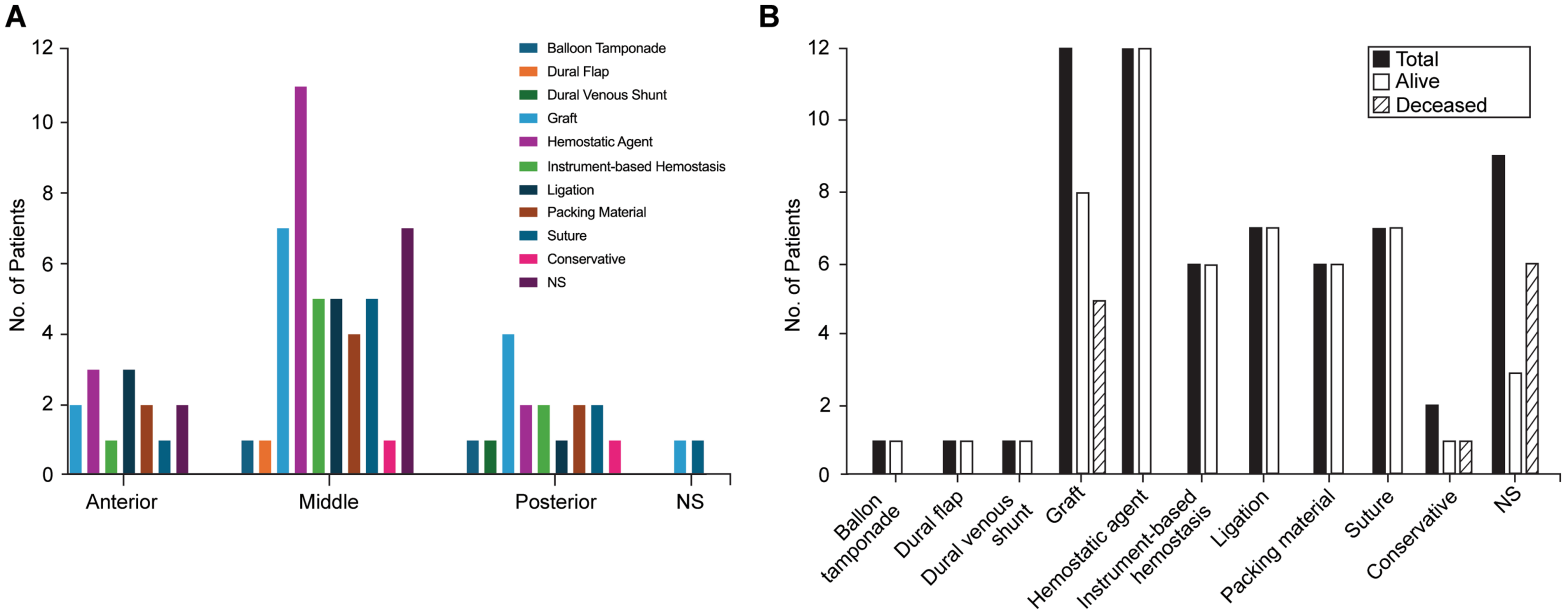
**(A)** Graph of bleeding control and repair methods across the 3 segments of the superior sagittal sinus (SSS). Cases that presented with an injury to 2 segments of the SSS were included in both sections of the graph. **(B)** Graph illustrating patient mortality with respect to the method of repair employed. NS, not specified. Used with permission from Barrow Neurological Institute, Phoenix, Arizona.

Repair strategies to the various SSS segments and their success rate varied by era. The most common mechanisms for bleeding control and sinus repair were grafts (e.g., autologous, synthetic) (*n* = 13; 25%) and the use of hemostatic agents (e.g., gelatin, Gelfoam, fibrin glue, Oxycel, Surgicel) (*n* = 13; 25%) (Figures 4A,B). Other methods employed were as follows: balloon tamponade for temporary bleeding control (*n* = 1; 2%), dural flap (n = 1; 2%), dural venous shunt (n = 1; 2%), instrument-based hemostasis (e.g., clips, forceps) (*n* = 6; 12%), ligation (*n* = 7; 14%), packing material (e.g., cottonoid patties, lint, gauze, muslin) (*n* = 6; 12%), sutures (*n* = 7; 14%), and conservative management (*n* = 2; 4%) (Table 2) (6, 16, 18, 21–55). In 12 cases, multiple methods were used in combination to repair the sinus and control the bleeding. Nine cases (18%) did not specify a repair mechanism (25–27, 31, 42, 44, 56), whereas 2 patients (4%) died before an attempt to repair the sinus was possible (26, 35). The postoperative length of stay was reported in 32 cases, with a median of 15 days (interquartile range, 7–32 days), underscoring the variability in patient recovery trajectories across the individual cases. Furthermore, the postdischarge follow-up period was reported in only 20 cases, with a median duration of 180 days (interquartile range, 73–365). However, few cases in the 19th and early 20th centuries documented such results, which may reflect a limited emphasis on extended patient follow-up in early surgical literature.

**Table 2:** Categorization of bleeding control and repair techniques.

Domain, technique	Definition
Direct repair	
Balloon tamponade	Temporary intraluminal inflation of a balloon to compress the defect and control bleeding until definitive repair
Hemostatic agents (gelatin sponge, Gelfoam, fibrin glue, Oxycel, Surgicel)	Application of topical substances to promote clotting and stabilize bleeding surfaces
Instrument-based hemostasis (clips, forceps)	Mechanical occlusion of bleeding points or lacerations using surgical instruments
Packing material (cottonoid patties, lint, gauze, muslin)	Insertion of absorbent material to provide tamponade and achieve hemostasis, often as a temporary or adjunctive measure
Sutures	Primary closure of the sinus wall defect with stitches to restore continuity and preserve venous flow
Reconstruction	
Dural flap	Rotation or advancement of native dura to cover the sinus defect and reconstitute venous flow
Dural venous shunt	Placement of an intraluminal conduit to maintain venous drainage when the sinus lumen is compromised
Grafts (autologous vein, fascia, muscle, synthetic substitutes)	Use of biological or synthetic material to replace or reinforce sinus wall defects and restore venous patency
Ligation	
Sinus ligation	Surgical tying or excision of the sinus to control hemorrhage, sacrificing venous flow; reserved for selected cases with sufficient collateral drainage
Other	
Conservative management	Nonoperative treatment (e.g., observation, medical stabilization) without direct surgical intervention

Internal categorization of bleeding control and repair techniques for penetrating injuries of the superior sagittal sinus. These domains and classifications were developed solely to structure the present review and to support logical analysis, rather than to represent established or official categories.

### 3.5 Neurological status

Neurological status reporting shifted from narrative descriptions in the 19^th^ and 20^th^ centuries to the use of the GCS by 2009. At the time of admission, patients exhibited a wide spectrum of neurological status, ranging from unconsciousness to alert with no deficits, with intermediate findings including impaired consciousness (e.g., incoherence, apathy, loss of inhibition) and focal deficits (e.g., spastic paraplegia, hemiplegia, hemihypoesthesia) (16, 26, 27, 29, 31, 33). Three patients were noted to have a neurological status indicative of a “longitudinal sinus syndrome,” characterized by bilateral leg and proximal arm weakness with early rigidity from venous obstruction (25, 26). Initial GCS scores were reported in 16 cases, beginning in the year 2009, with a mean (SD) of 11.8 (3.8). Of these 16 patients, 2 had severe TBIs (GCS 3–8), 7 had moderate TBIs (GCS 9–13), and 7 had mild TBIs (GCS 14–15) (60). Finally, 1 patient was reported to have lost his pulse upon arrival and was pronounced dead soon after (35).

### 3.6 Outcome

Of the 51 cases reviewed, 37 patients (73%) survived their injuries (Table 1). The percentage of survivors varied across time periods. In the 19th century, all reported cases survived (5/5). However, between 1900 and 1949, survival decreased to 29% (4/14). In the latter half of the 20th century, survival increased to 88% (7/8), and in contemporary reports (2000–present), survival was 88% (21/24) (Table 1). Among the survivors, 23 (62%) experienced no neurological deficits postoperatively. Eight patients (22%) were left with mild residual symptoms such as paresthesia or slight visual disturbances, and 6 patients (16%) sustained significant neurological deficits, including hemiparesis and visual loss (Table 1). Of the 14 patients (27%) who succumbed to their injuries, 4 patients had injuries in the anterior segment (26, 27, 56), 8 in the middle (26, 27, 42, 45), and 1 in the posterior (26), with 1 deceased patient (7.1%) having an unspecified SSS segmentation (Figure 3C) (35). Of the 11 deceased patients who underwent repair, 5 received grafts (26, 27); the technique employed was not specified for the remaining 6 patients (Figure 4B) (26, 27, 42, 56). One deceased patient received only conservative management (45), whereas 2 died before surgical repair could be attempted (26, 35). Mortality was highest for patients with anterior lesions (4 of 12), followed by middle (8 of 34) and posterior lesions (1 of 9), in comparison with the overall mortality of 27% (14 of 51).

## 4 Discussion

### 4.1 Anatomical considerations: thirds of the SSS

pSSSIs represent a formidable neurosurgical challenge, with outcomes shaped by both anatomical location and evolving management. The anterior third has long been considered the most forgiving (4, 61, 62), because it typically drains only a small portion of the frontal lobes (62). Collateral circulation often compensates for injury (3), a perception reinforced over a century ago when Edward Archibald (1872–1945) remarked that “a tear in that part [of the SSS] is said to be unimportant.” (61) However, this presumed safety is not absolute. In patients with a dominant anterior drainage or compromised collaterals, injury to this segment can result in venous infarction, edema, and even death (3, 62). Results of our study support this duality. In our review, 12 of 48 cases with specification (25%) involved the anterior third (Figure 3C), and 4 of these patients died. This is comparable to overall mortality (14 of 51; 27%), underscoring that anterior injuries can be lethal (61). Our results further support this, because anterior lesions appeared to be associated with the highest mortality, despite the limitations of a small sample size and incomplete reporting.

The middle third was most frequently affected, involved in 34 of 48 cases (71%) (Figure 3C). This segment drains much of the cerebral hemispheres and is a major venous outflow pathway (1, 63), which may explain why 8 of 14 total deaths occurred here. Its central role and high flow make repair both critical and technically demanding, reflected in the wide variety of strategies attempted (Figure 4A) (1, 46, 64).

Posterior segment injuries were less common (9 of 48; 19%) but theoretically dangerous, given their role in draining parietal and occipital lobes into the confluence (65). In our analysis, only 1 patient died (Figure 3C), although the small sample size precludes definitive conclusions; further data are needed.

### 4.2 Surgical management strategies over time

#### 4.2.1 The 19th century

**Figure 5:**
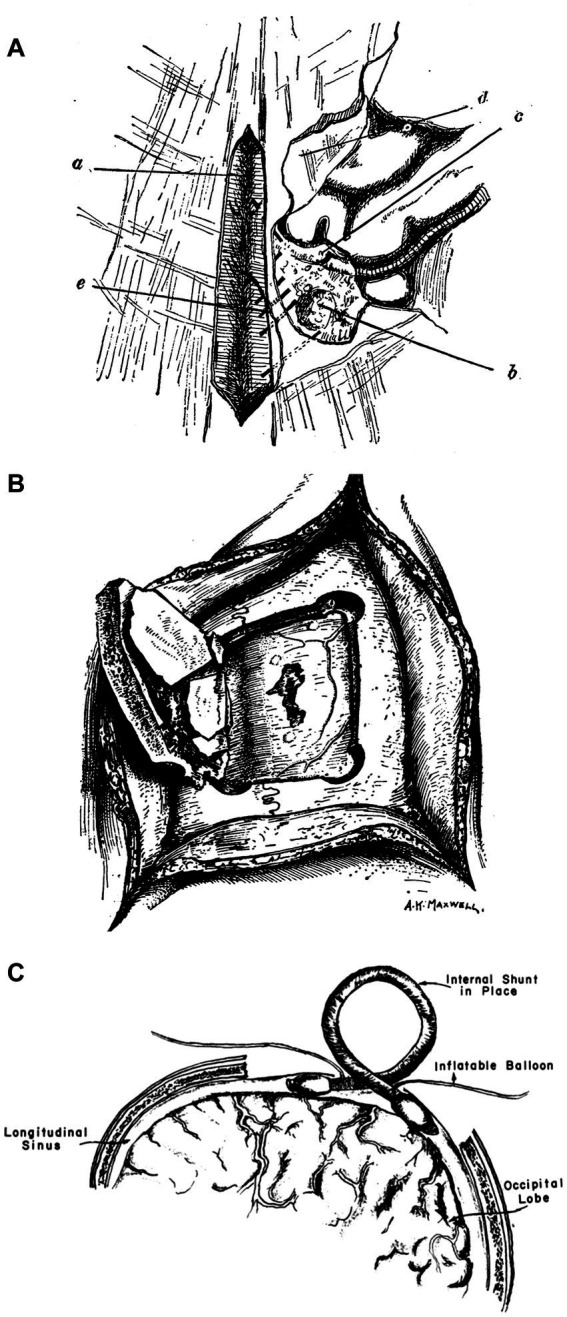
Historical illustrations of the superior sagittal sinus (SSS) and mechanisms of its repair. **(A)** Illustration taken from Holmes and Sargent’s 1915 work, Injuries of the Superior Longitudinal Sinus (25). (*a*) SSS opened, (*b*) lateral lacuna with Pacchionian tuft (arachnoid granulation projecting into the sinus), (*c*) valve-like opening of a cortical vein, (*d*) dura reflected to show brain surface, and (*e*) glass rods showing venous channels from the lacuna to the sinus. Reproduced from British Medical Journal, Holmes G, Sargent P, vol 2(2857), pp 493–498, 1915, with permission from BMJ Publishing Group Ltd. **(B)** Image taken from Harvey Cushing’s 1918 work, Notes on Penetrating Wounds of the Brain (66), depicting the elevation of a depressed skull fracture from the mid-vertex, revealing a dural laceration with a tear along the margin of the SSS. Reproduced from British Medical Journal, Cushing H, vol. 1(2982), pp 221-226, 1918, with permission from BMJ Publishing Group Ltd (66). **(C)** Schematic taken from John P. Kapp’s 1971 work, An Internal Shunt for Use in the Reconstruction of Dural Venous Sinuses (28). The illustration describes an internal dural sinus shunt made of a modified pediatric endotracheal tube with inflatable cuffs to maintain venous flow and provide hemostasis during the sinus repair. From Kapp et al., An internal shunt for use in the reconstruction of dural venous sinuses. Technical note, J Neurosurg, 1971. Used with permission from the JNS Publishing Group. An Open Access or Creative Commons publishing model conveys no rights to use this material in any format without written permission from the JNS Publishing Group.

The approach to repairing pSSSIs has undergone a refinement over the past 2 centuries, expanding from simple packing to sutures, grafts, and advanced hemostatic agents, many of which remain in use today (Figure 5) (25, 28, 66). To our knowledge, the earliest reported case detailing the repair of a pSSSI was of a 44-year-old Englishman who was thrown from his horse in late 1826 (16). After seeking refuge at a distant cottage, the man was found to have sustained a severe fracture, with a large fragment of bone thrust into his sinus. Hemorrhaging profusely, the sinus was promptly attended to “by the introduction of a plug of lint” (16). This case exemplifies the beginnings of approaching a venous sinus bleed: simple packing, applied with urgency. Nearly 60 years later, William Hopkins (1853–1904) confronted a similar case. However, despite repeated attempts to suture the clearly exposed wound, he ultimately resorted to finger pressure and a lint compress (22). These early reports demonstrate how surgeons recognized the need for vascular repair; however, without modern techniques, even the most straightforward repairs often resulted in packing as the only dependable option.

#### 4.2.2 Dawn of neurosurgery

By the 1890s, surgeons began experimenting with more deliberate hemostasis. William Keen (1837–1932), operating on a patient struck by a falling wheel, described a compound fracture with 2 rents in the sinus (24). He achieved control using forceps for one tear and iodoform gauze for the other, leaving the forceps in place for 2 days without incident—a reflection of the pragmatic boldness characteristic of early neurosurgeons.

In 1908, Harvey Cushing (1869–1939) declared that a pSSSI “can only be packed in order to control hemorrhage.” (67) However, this did not impede Henry Gray (1870–1938) from exploring the “postage stamp” technique in 1916, involving a fascial graft applied under direct pressure over sinus defects, cautioning that such repairs were not to be attempted by the inexperienced due to the risk of “alarming hemorrhage.” (68) Despite his prior remarks, Cushing used autologous grafts and clips for sinus repair the following year in his seminal manuscript on military head wounds (26, 66). The early 1900s culminated with Harold Neuhof (1884–1964) advocating a graded approach to pSSSIs: grafts for small defects, sutures or clips for larger tears, and ligation for transections (69). These examples mark the transition from improvised packing to structured repair strategies.

#### 4.2.3 Late 1900s

Reports were sparse in the mid-century, but the 1970s introduced significant advances (28, 30, 33). Kapp et al. (28) constructed an internal shunt from a modified pediatric endotracheal tube (Figure 5). In their illustrative case, a shunt was temporarily inserted into the SSS of a patient with a penetrating occipital injury, enabling controlled reconstruction with an autologous saphenous vein graft. This innovation addressed bleeding control, visualization, and venous congestion, representing one of the earliest documented uses of both sinus shunts and autologous vein grafts for SSS repair (28).

Baurand et al. (70) reported a case series of 20 traumatic dural venous sinus injuries and primarily contributed conceptual insights into injury patterns and early management. A key observation was that many sinus wounds were initially controlled by spontaneous or fracture-mediated tamponade, often permitting delayed operative intervention under more controlled circumstances. The authors emphasized that depressed bone fragments frequently both create and temporarily seal the sinus defect, a mechanism they proposed as an explanation for the relatively low rate of acute hemorrhagic complications observed in their series (70).

Hassler (56) reported a single-center series whose primary contribution was a technically focused discussion of operative management for dural venous sinus injuries, detailing strategies such as direct suturing, patch grafting, clip application, and autologous vein reconstruction, along with practical intraoperative principles for hemorrhage control and thrombosis prevention. A central point emphasized was that depressed skull fractures often both create and temporarily tamponade sinus lacerations, with displaced bone fragments providing intrinsic hemostasis. The authors also underscored the prognostic and operative importance of injury topography, noting the relatively greater tolerance of anterior third injuries compared with the higher risk associated with more posterior segments (56).

Concurrently, hemostatic agents such as Gelfoam and Surgicel gained popularity, providing reliable local control and supplementing or replacing older methods such as packing or digital pressure (30, 32, 33). Although not altering repair strategies, they improved intraoperative safety and efficiency.

#### 4.2.4 Contemporary approaches

In recent decades, repair strategies for pSSSIs have evolved into a more deliberate and structured set of options. Although the literature remains limited and often anecdotal, for a better understanding, the techniques available were broadly categorized into 3 groups within our study: direct repair, reconstructive methods, and ligation (Table 2).

**Figure 6:**
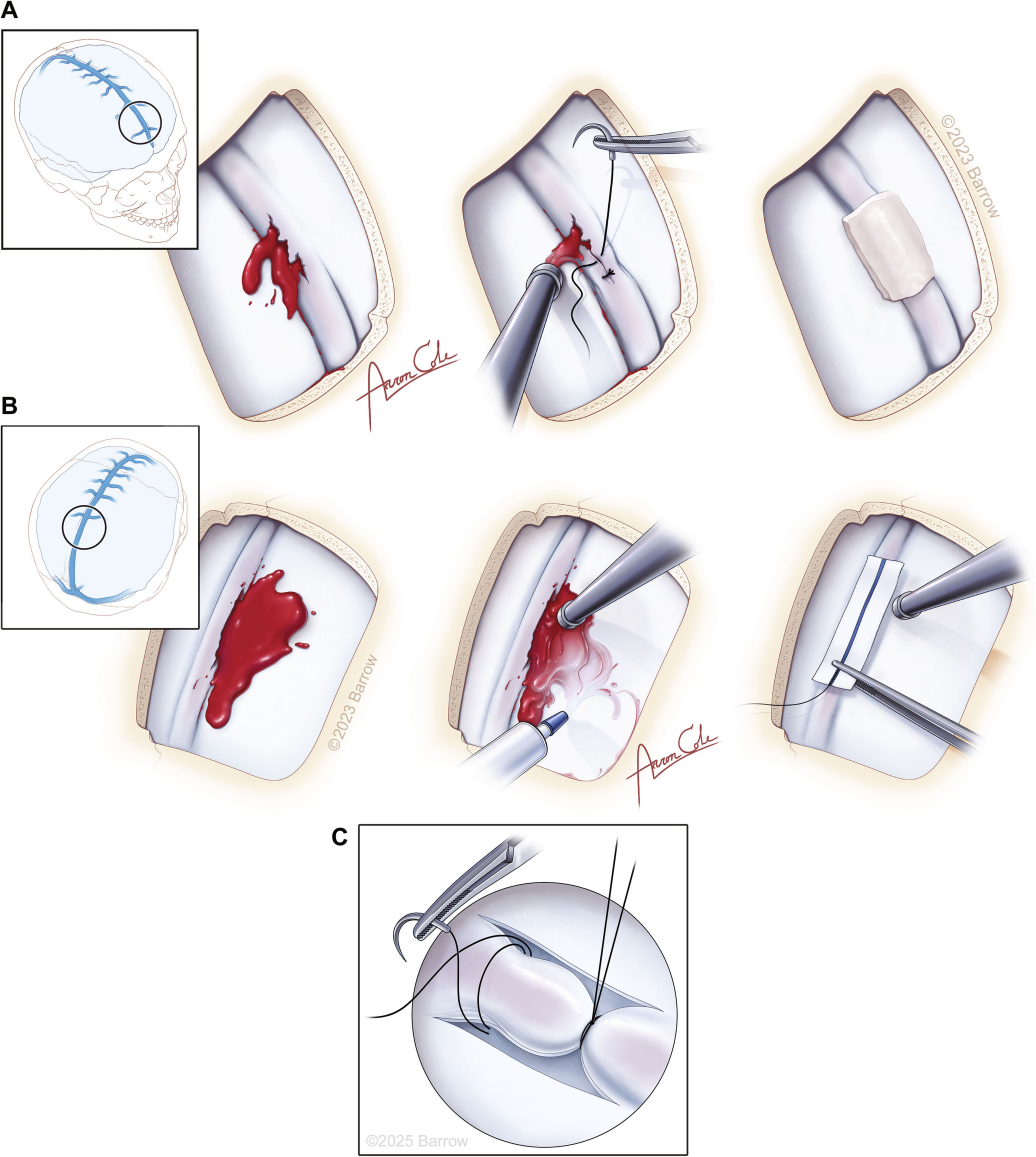
Illustrations depicting simple techniques for repair of a penetrating injury of the superior sagittal sinus. **(A)** Direct suturing. **(B)** Use of a hemostatic agent. **(C)** Ligation. Used with permission from Barrow Neurological Institute, Phoenix, Arizona.

Direct repair encompasses suture closure, clips, packing (e.g., cottonoid patties, gauze), and hemostatic agents such as Gelfoam, Surgicel, gelatin sponges, and fibrin glue (Figure 6). Although packing material continues to be used in some cases, echoing a lineage of early interventions, it is now often used in tandem with other techniques rather than as a standalone method (47, 51).

**Figure 7:**
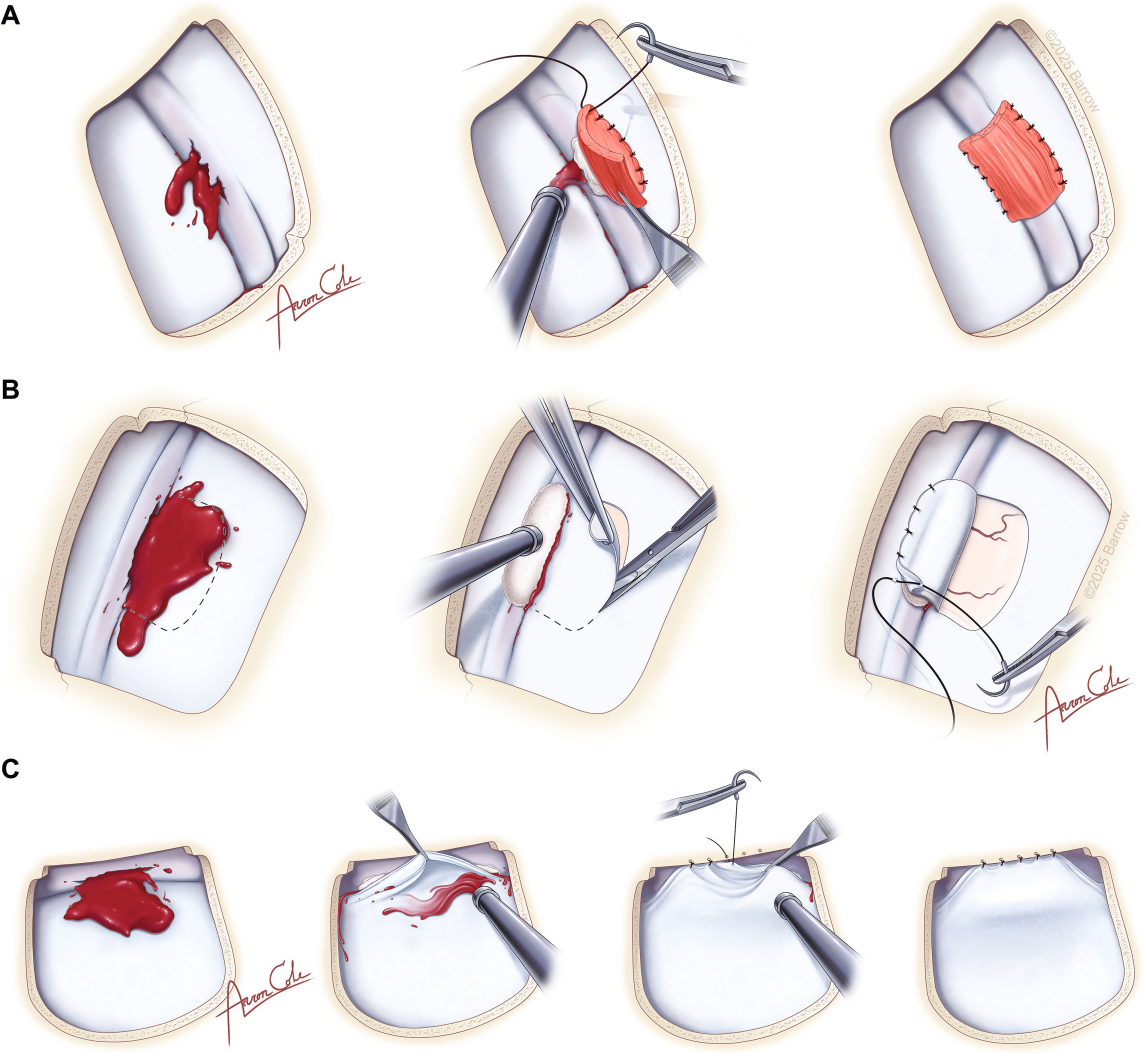
Illustrations demonstrating the repair of penetrating injuries of the superior sagittal sinus through reconstructive methods. **(A)** Autologous muscle grafting. **(B)** Dural flaps. **(C)** Dural tenting. Used with permission from Barrow Neurological Institute, Phoenix, Arizona.

Reconstructive techniques are typically reserved for larger defects or disrupted sinus walls. These include autologous vein, fascia, or muscle grafts, synthetic substitutes, and dural flaps (Figures 7A,B) (48). Hybrid strategies are increasingly common, such as pairing grafts and flaps with hemostatic overlays or using balloon tamponade as a temporary measure to facilitate a more controlled suture closure (46, 48, 51, 52). Suture tack-ups, akin to dural tenting, were also used to aid in hemostasis (Figure 7C) (55).

Interestingly, despite its alluded use in other SSS injuries, ligation was not documented in our collection of pSSSI literature until recently (Figure 6) (38). In 1924, Cushing suggested that “a large section” of the anterior third of the sinus could be excised safely when involved by a tumor, echoing Archibald’s ideas of its unimportance (4, 61). However, nearly 100 years later, in 2012, a study focusing on anterior third ligation in meningioma surgery warned that “ligation of the unoccluded [anterior SSS] will produce severe blood flow disturbances in some cases, leading to cerebral edema and venous infarct,” and concluded that such a maneuver “should be avoided as far as possible” unless preoperative venography confirmed robust collateral drainage (62).

Regardless of this warning, ligation appeared 7 times in our review between 2012 and 2025, including 2 cases reported in 2025 alone (41, 43, 49, 50, 53, 54). Notably, none of the 7 patients who underwent ligation died. However, complications such as cognitive deficits and intracranial hypertension were noted (41, 53), an outcome consistent with the concerns about cerebral edema (62). These results suggest ligation may be better tolerated than previously assumed, provided collateral drainage is sufficient.

### 4.3 Repair strategies based on the extent of the injury

**Figure 8:**
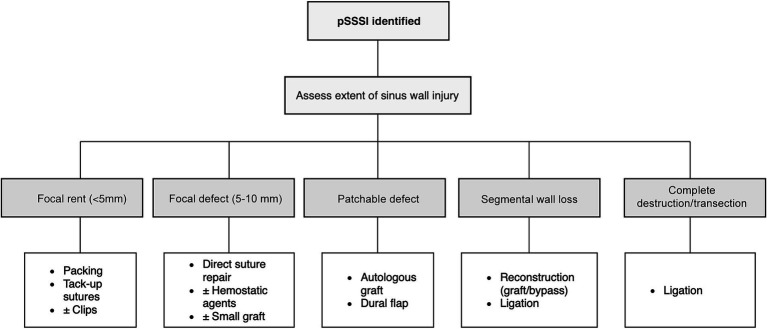
Flowchart showing strategies for management of penetrating injury of the superior sagittal sinus (pSSSI) according to the extent of sinus wall injury. Used with permission from Barrow Neurological Institute, Phoenix, Arizona.

Although techniques can be grouped into the broad categories of direct repair, reconstruction, and ligation (Table 2), a complementary way of understanding surgical decision-making is to consider the extent of the sinus wall injury (Table 3). Such categories could include focal rents (<5 mm), focal wall defects (5–10 mm), patchable wall loss, segmental or destructive wall loss, and complete destruction or transection (Figure 8).

**Table 3:** Treatments of penetrating injuries of the superior sagittal sinus.

Segment, penetrating object	Extent of injury	Successful treatment	Unsuccessful treatment	Outcome
Anterior				
Bone fragment (16)	Focal rent	Plug of lint	NA	Alive
Bone fragment (26)	Focal rent (laceration to right of midline, controlled easily)	Silver clip	NA	Alive
Bone fragment (27)	Focal rents (multiple)	Muscle grafts	NA	Deceased: septic meningitis and frontal abscess, not direct SSS cause
Bullet (27)	Focal rents (through and through punctures)	Muscle grafts	NA	Deceased: meningitis and ventriculitis (ventricular pus, purulent exudate encircling brainstem/cord)
Bullet (56)	Segmental/destructive wall loss (4-cm laceration)	NS	NA	Deceased: unknown cause
Nail (47)	Focal wall defects (entry and exit sites from 2 nails)	Packing with foam-based hemostatic products; cottonoid patties	NA	Alive
Nail (50)	Segmental/destructive wall loss (15-mm segment punctured by nail, filling defect and AV shunt)	Sinus ligation anterior and posterior to puncture site; nail removal	NA	Alive
Bolt gun rod (53)	Complete transection	Ligation	NA	Alive
NS (26)	Complete destruction	NA	Bleeding control attempts (details not specified)	Deceased: secondary hemorrhage, infection (encephalitis, meningitis, ventriculitis)
Tile fragment (37)	Focal wall defect (penetration just anterior to coronal suture)	Removal of foreign body; hemostasis with Gelfoam, Surgicel, and pressure	NA	Alive
Anterior and middle				
Bone fragment (40)	Focal wall defect (partial sinus tear at vertex)	Elevation of depressed fragments; gelatin foam seal	NA	Alive
Knife (54)	Complete transection	Ligation of both ends of SSS with silk sutures; aneurysm clips for proximal and distal control	Attempted anastomosis (not feasible due to excessive tension)	Alive
Middle				
Bone fragment (27)	Focal rent (from indriven bone fragment)	NA	Bleeding control attempts (details not specified)	Deceased: meningitis and encephalitis due to infection (*Bacillus welchii*), not direct SSS cause
Bone fragment (27)	Focal rent (puncture by bone fragment)	NA	Muscle graft	Deceased: meningitis/encephalitis with secondary ventricular involvement, not direct SSS cause
Bone fragment (22)	Focal wall defect (circular hole in sinus from bone fragment, ~1/16 inch)	Compress of lint with iodoform	Ligature attempts (failed repeatedly)	Alive
Bullet (27)	Focal rent	NA	Bleeding control attempts (details not specified)	Deceased: meningitis and ventriculitis (*B. welchii* infection, purulent fluid in ventricle), not direct SSS cause
Bullet (31)	Focal rent/possible laceration along missile tract (minimal bleeding)	Bleeding controlled with debridement/irrigation (no formal repair required)	NA	Alive
Bullet (25)	Segmental/destructive wall loss (bullet traversed midline with comminuted fracture and sinus involvement)	NS	NS	Alive
Bullet (45)	Segmental/destructive wall loss (extensive laceration with tissue destruction)	NA	RNA	Deceased: pulmonary air embolism and severe traumatic brain injury
Bullet (41)	Segmental/destructive wall loss (tangential GSW with sinus disruption at vertex)	Ligation	NA	Alive
Knife (39)	Focal wall defect (knife penetration with marginal sinus bleeding)	Sinus leak sealed with autologous material	NA	Alive
Nail (33)	Focal rent	Gelfoam; external pressure	NA	Alive
Nail (33)	Focal rent (1-cm laceration)	Gelfoam; figure-8 dural sutures	NA	Alive
Nail (46)	Focal wall defect	Nail removal; temporary vein clip; hemostatic packing; dural flap repair with sutures; synthetic dural cover	NA	Alive
Nail (52)	Focal wall defect	Gore-Tex patch; gelatin sponge; fibrin glue	NA	Alive
Nail (32)	Focal wall defect (nail puncture at vertex)	Nail removal under direct inspection; hemostasis with Oxycel	NA	Alive
Nail (18)	Focal wall defect (nail puncture through both leaflets)	Nail removal under direct vision; primary suture repair of both inner and superficial SSS leaflets	NA	Alive
Nail (30)	Focal wall defect (nail puncture through sinus wall)	Nail extraction; hemostasis with Surgicel and pressure	NA	Alive
Nail (34)	Patchable wall loss (large hole in sinus wall)	Temporal muscle/fascia graft secured with suture traction and bone wax	NA	Alive
Nail (38)	Segmental/destructive wall loss (through-and-through sinus injury)	Sinus ligation anterior and posterior to nail	NA	Alive
Shrapnel (26)	Focal rent (bleeding from sinus during bone fragment removal, controlled)	Fascial graft	NA	Deceased: inanition (secondary to bedridden hemiplegia); not direct SSS cause
Shrapnel (26)	Focal wall defect	Silver clips ×3 on sinus margin	NA	Alive
Shrapnel (26)	Segmental/destructive wall loss	NS	NS	Deceased
Coin (49)	Segmental/destructive wall loss (~3-cm rupture, thrombosed ends)	Ligation of rostral and dorsal sinus orifices; fibrin glue; dural patch	NA	Alive
Iron pipe (42)	Segmental/destructive wall loss (4-cm complex longitudinal laceration)	NA	Bleeding control attempts (details not specified)	Deceased: exsanguination from SSS injury (hypovolemic shock)
Marble fragment (36)	Focal wall defect (with compression/obstruction)	Initial hemostasis and closure after bone fragment removal; second operation: removal of compressing stone, Surgicel for sinus wall bleeding, dural repair with fascial graft	NA	Alive
NS (26)	Segmental/destructive wall loss (irregular tear of sinus wall with anastomotica magna vein torn)	NA	RNA (no operation performed before death)	Deceased: direct SSS injury with thrombosis and massive cerebral disruption
Rake tooth (51)	Patchable wall loss	MacKenzie clips; packing material; pericranial dural patch	NA	Alive
Screw (44)	Focal rents (screws penetrating sinus and falx)	Screw removal under direct visualization with craniectomy exposure; proximal/distal sinus control achieved (no bleeding observed)	NA	Alive
Middle and posterior				
Bone fragment (24)	Patchable wall loss (2 rents in sinus: 1 large, 1 small)	Hemostatic forceps applied to large rent; iodoform gauze packing to small rent	NA	Alive
Nail (55)	Focal rents (multiple nail punctures of SSS)	Nail removal; Surgicel; gentle pressure; dural tack-up sutures	NA	Alive
Shrapnel (48)	Segmental/destructive wall loss	PTFE vascular graft with 6–0 polypropylene sutures; balloon tamponade (auxiliary, temporary control); vascular clamps for exposure	NA	Alive
Drill bit (43)	Complete destruction	Ligation	NA	Alive
Tree branch (21)	Patchable wall loss (fracture and depression with sinus wall tear)	Hemostasis with compress of muslin applied over sinus; bone removal and elevation of depressed fragments	NA	Alive
Posterior				
Knife (6)	Segmental/destructive wall loss	Dural repair with periosteal graft; removal of bone fragments; RNA (sinus repair not attempted)	NA	Alive
Shrapnel (26)	Focal wall defect (sinus laceration at occipital gutter)	Muscle grafts ×2 (hemostasis achieved)	NA	Deceased: meningitis/brain infection
Shrapnel (28)	Patchable wall loss (outer leaf 2-cm and right lateral wall 1.5-cm lacerations)	Balloon shunt for temporary control; primary repair of right lateral wall (continuous suture); saphenous vein patch graft to outer leaf	NA	Alive
Shrapnel (29)	Patchable wall loss (penetrating defect just proximal to torcular Herophili)	Muscle stamp (partially inserted into sinus, hemostatic and effective); primary closure of dura/wound	NA	Alive
NS				
Bullet (27)	Focal rents (2 sites)	Muscle grafts (autologous, from patient’s leg)	NA	Alive
Iron spike (23)	Patchable wall loss (plugged by bone fragment, profuse bleeding after removal)	Primary suture repair (catgut)	NA	Alive
Saw blade (35)	Segmental/destructive wall loss (long full-thickness sinus and skull laceration with brain loss)	NA (died before intervention)	NA (died before intervention)	Deceased: exsanguination and massive traumatic brain injury

AV, arteriovenous; GSW, gunshot wound; NA, not applicable; NS, not specified; PTFE, polytetrafluoroethylene; RNA, repair not attempted; SSS, superior sagittal sinus.

At the most limited end of the spectrum, focal rents were typically managed with relatively simple measures such as packing (lint, Gelfoam, Surgicel), tack-up or figure-eight sutures, small clips, or fascial grafts. Mortality in this group was largely attributable to secondary infection rather than exsanguination, highlighting that technical control of bleeding was usually successful, but postoperative complications dictated outcomes. A step further, focal wall defects required direct repair or reinforcement using sutures, grafts, and adjunctive hemostatic materials (Table 3) (16, 26, 27, 47, 50, 56). These approaches were highly effective, and survival rates were excellent, with the rare fatalities again tied to infection rather than intraoperative failure.

Patchable lacerations were uniformly survivable, with techniques including autologous grafting (muscle, fascia, pericranium, saphenous vein), frequently secured with sutures and supported by temporary adjuncts such as clips or balloon shunts (Table 3). This group displayed the most consistent success, underscoring the effectiveness of patch reconstruction when the defect is limited enough to permit coverage. By contrast, segmental or destructive wall loss posed a much greater operative challenge. Survival was achieved in some patients through proximal and distal ligation or formal vascular reconstruction with grafts and patches. In others, uncontrolled bleeding or the inability to perform a definitive repair led to death from massive exsanguination (Table 3).

At the most catastrophic end of the spectrum, cases of complete destruction or transection demonstrated that survival was only reliably achieved through ligation of the sinus, with or without adjunctive clip control (Table 3). Attempts at anastomosis or nonspecific hemostatic maneuvers uniformly failed, reflecting the limits of reconstructive potential in this scenario.

When viewed this way, a graded trend in pSSSI repair emerges, not unlike Neuhof’s proposal over 100 years ago (69). Limited injuries (e.g., rents, focal wall defects) are typically managed with simple hemostatic or reconstructive measures, with intermediate lesions responding well to structured patch repair. More extensive injuries with segmental loss may be salvaged with ligation or graft-based reconstruction, whereas complete destruction or transection leaves ligation as the only reliable option. Repair complexity escalates in step with the extent of sinus wall injury, providing a logical basis for operative strategy.

### 4.4 Outcomes and common patterns

Overall mortality was 27% (14 of 51), with anterior-segment injuries demonstrating the highest mortality rate and middle-segment injuries accounting for the greatest number of deaths (Figure 3C) (1, 63). Interestingly, no multisegment injuries proved fatal. Ligation was typically reserved for complex or irreparable injuries, and outcomes were variable, often reflecting the severity of the underlying trauma rather than the procedure itself (38, 41, 43, 49, 50, 53, 54). Autologous grafts were associated with high mortality (5 of 13 patients), although all deaths occurred before 1920 (Figure 4B) (26, 27). These early outcomes likely reflect historical confounders rather than the reconstructive technique itself, including infection, wartime conditions, and limited perioperative care, particularly in the preantibiotic era.

Consistent with this, the available data do not demonstrate a clear, linear improvement in survival over time. Survival appears high in the 19th century; however, this is based on a small number of reported cases (5/5 cases), limiting meaningful interpretation. A decline is observed in the early 20th century (1900–1949; 4/14, 29%), followed by higher reported survival in later periods (e.g., 7/8 after [88%] 1950 and 21/24 [88%] after 2000) (Table 1). Although modern-era reports may reflect improved outcomes, particularly relative to early 20^th^ century case reports, these comparisons are limited by small sample sizes and a likely selection toward survivable cases.

**Figure 9:**
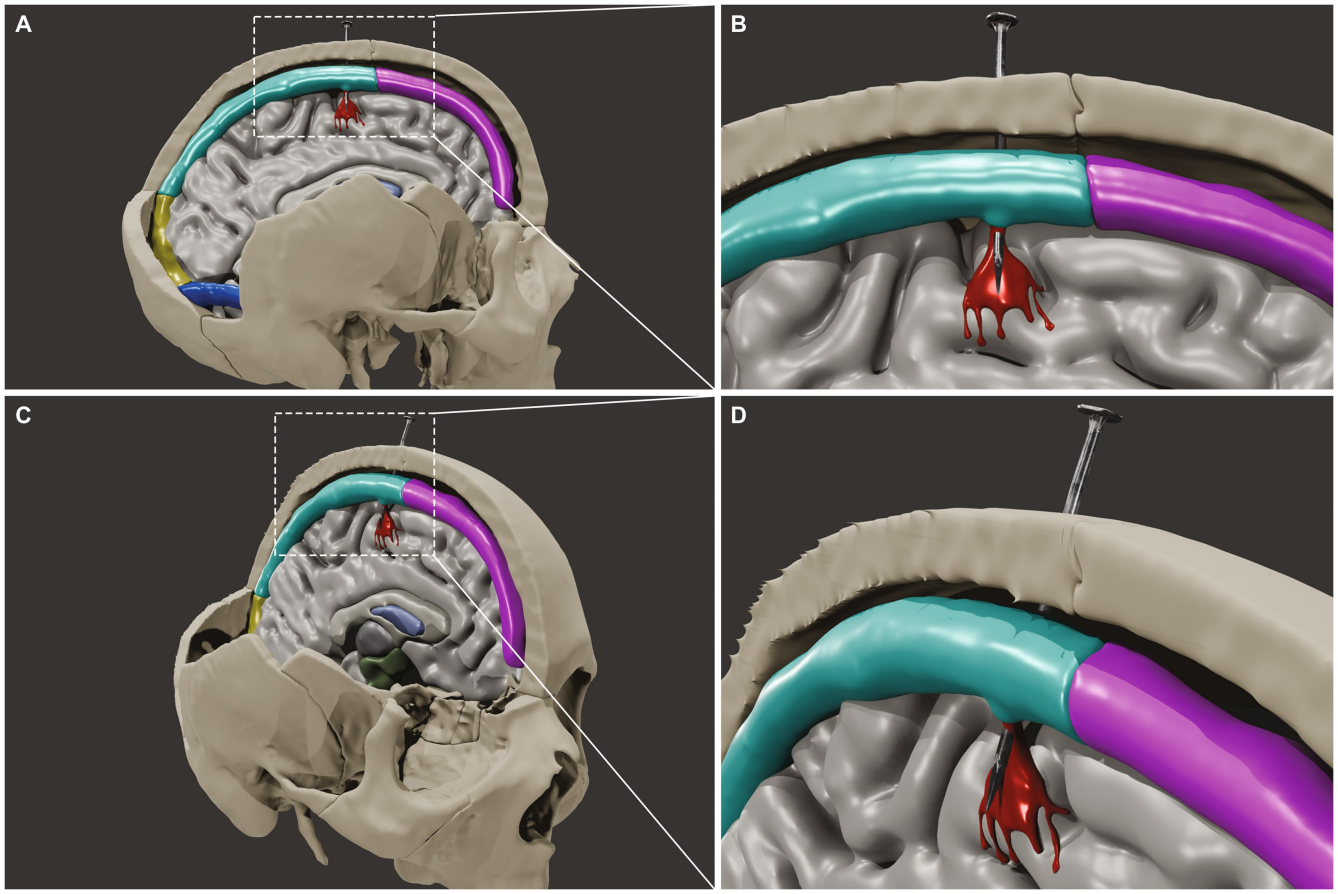
Three-dimensional illustrations depicting a nail injury in the middle third of the superior sagittal sinus (SSS). The SSS is shown divided into anterior (*purple*), middle (*turquoise*), and posterior (*yellow*) segments. **(A)** Lateral view depicting the penetrating nail injury. **(B)** Detail of the lateral view. **(C)** Oblique view depicting the penetrating nail injury. **(D)** Detail of the oblique view. Models were created through segmentations in 3D Slicer (https://www.slicer.org/) and then visualized in Blender (https://www.blender.org/). The nail model was obtained from Sketchfab (https://sketchfab.com/3d-models/nail-old-nail-with-rust-efc482ed6eba4d3a9baac1a7528b9372) originally created by uncledima_official, and is used under the Creative Commons Attribution (CC BY) license.

**Figure 10:**
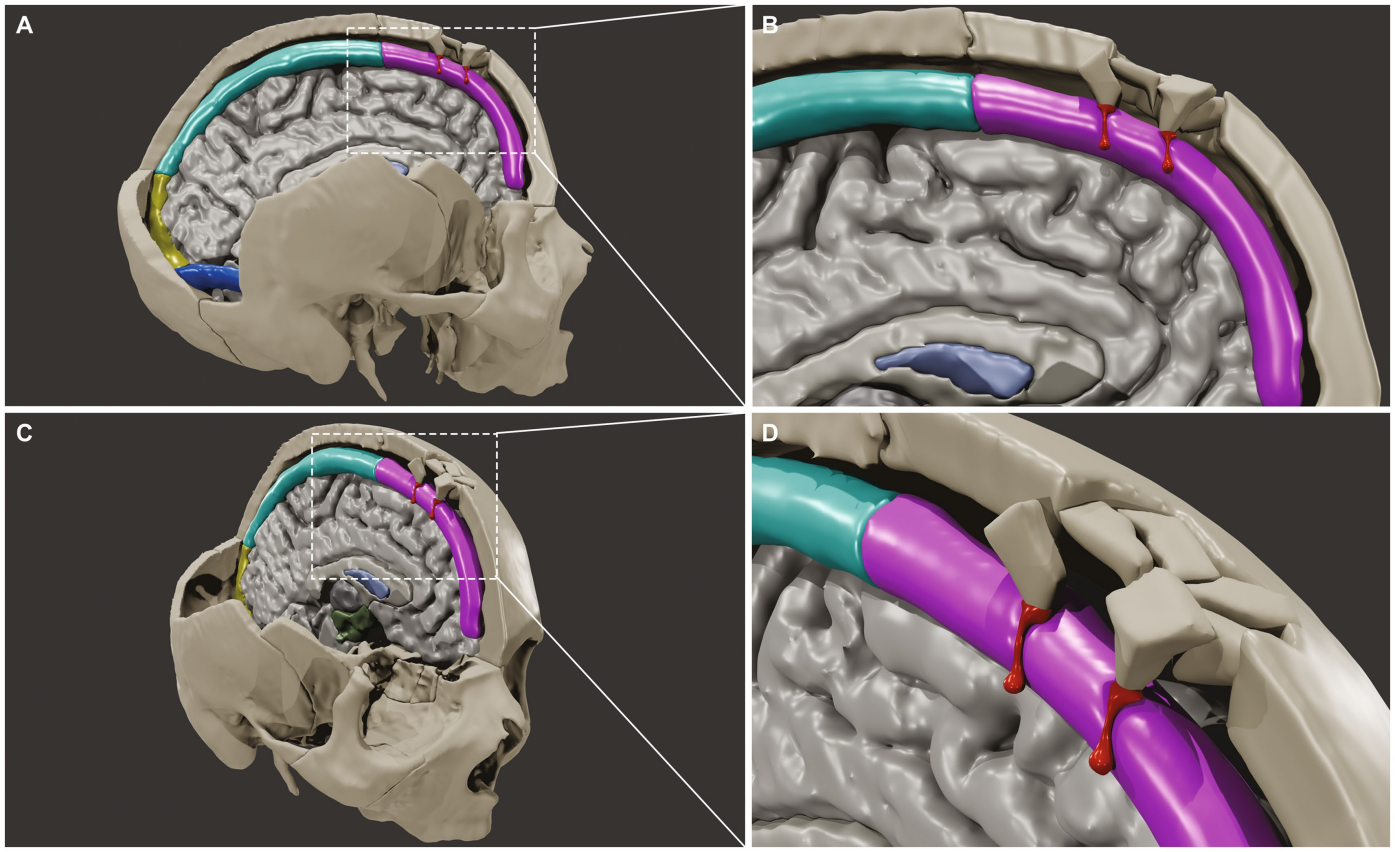
Three-dimensional reconstruction illustrating a decompressive skull fracture penetrating into the anterior third of the superior sagittal sinus (SSS). The SSS is shown divided into anterior (*purple*), middle (*turquoise*), and posterior (*yellow*) segments. **(A)** Lateral view of the skull fracture. **(B)** Detail of the lateral view. **(C)** Oblique view of the skull fracture. **(D)** Detail of the oblique view. Models were created through segmentations in 3D Slicer (https://www.slicer.org/) and then visualized in Blender (https://www.blender.org/).

The injury mechanism also appeared to influence mortality. Nails, the most common penetrating object, as well as knives, generally produced cleaner trajectories and more isolated injuries, often lending themselves to simpler repairs and better outcomes (Table 1; Figure 9). In contrast, bullets, shrapnel, and bone fragments, with their rougher injury mechanics and more extensive damage, were associated with poorer neurologic status and a higher mortality (Figure 10). Clinical status at admission was not always predictive; patients with poor initial examination findings sometimes recovered (26, 30, 31), whereas others experienced deterioration despite initial stability (24, 32, 51).

### 4.5 Limitations of the included studies and future directions

Because pSSSIs are rare, evidence is limited to case reports and small series, which carry risks of publication and selection biases. Immediately fatal or otherwise unreported cases likely exist, and some published examples may have been inadvertently omitted despite a comprehensive search strategy. Moreover, many reports focused on the broader context of trauma, providing limited sinus-specific detail. Additionally, temporal comparisons are confounded by advancements in perioperative care, including the introduction of antibiotics, which may influence observed survival trends independent of surgical technique. Although heterogeneity precluded formal meta-analysis, to our knowledge, this study offers the most comprehensive account to date, delineating injury patterns, management strategies, and outcomes and providing a foundation for future collaborative reporting and registry development in sinus repair after penetrating injuries.

Additionally, the limited literature on endovascular management of pSSSIs and penetrating injuries to other dural venous sinuses suggests a clear gap for future investigation and treatment technique development. Further work should also examine how emerging technologies, including artificial intelligence tools increasingly integrated into neurotrauma practice, might support treatment stratification and algorithm development for these injuries (71, 72). As new techniques evolve, iterative refinement and systematic evaluation of current approaches will be essential to improve safety and outcomes.

## 5 Conclusion

pSSSIs remain among the most formidable challenges in neurosurgery, demanding rapid judgment in anatomically complex and high-stakes scenarios. Despite technical advances, sinus repair remains an inherently high-risk procedure. Thin walls, high-flow venous pressure, and the central location of SSS allow little margin for error, with hemorrhage, thrombosis, and infarction ever-present risks. Even today, each case requires a careful balance between control and catastrophe. However, it seems there is direct benefit in aggressive management, revealed by the fact that almost two-thirds of pSSSI survivors showed no neurological deficits postoperatively, and nearly 20% of survivors had only mild neurological deficits. Although no universal protocol exists, patterns are emerging, shaped by the extent of the injury and historical choices of intervention.

## Data Availability

The original contributions presented in the study are included in the article/Supplementary material, further inquiries can be directed to the corresponding author/s.
