# Supplementary material for: A systematic review of penetrating injuries to the superior sagittal sinus across two centuries
Source: Front Neurol. 2026 Jun 11;17:1789736. doi: 10.3389/fneur.2026.1789736 (PMC13295164; doi:10.3389/fneur.2026.1789736)
Supplement: Supplementary file 1 [file Supplementary_file_1.docx]

**SUPPLEMENTAL MATERIALS**

**Supplemental Table 1.** Joanna Briggs Institute risk of bias quality assessment

| **Author(s)** | **Q1** | **Q2** | **Q3** | **Q4** | **Q5** | **Q6** | **Q7** | **Q8** | **Q9** | **Q10** | **Rate (%)** | **Risk** |
| --- | --- | --- | --- | --- | --- | --- | --- | --- | --- | --- | --- | --- |
| Abdallah et al., 2022([48](#_ENREF_48)) | Yes | Yes | Yes | Yes | Yes | Yes | Yes | Yes | — | — | 100 | Low |
| Arham & Zaragita, 2021([46](#_ENREF_46)) | Yes | Yes | Yes | Yes | Yes | Yes | Yes | Yes | — | — | 100 | Low |
| Baig et al., 2025([54](#_ENREF_54)) | Yes | No | Yes | Yes | Yes | Yes | No | Yes | — | — | 75 | Low |
| Balak et al., 2009([36](#_ENREF_36)) | Yes | Yes | Yes | Yes | Yes | Yes | Yes | Yes | — | — | 100 | Low |
| Brisman & Harrington, 1973([29](#_ENREF_29)) | Yes | No | Yes | Yes | Yes | Yes | Yes | Yes | — | — | 87.5 | Low |
| Brune et al., 2018([45](#_ENREF_45)) | Yes | Yes | Yes | Yes | Yes | Yes | Yes | Yes | — | — | 100 | Low |
| Cole, 1849([21](#_ENREF_21)) | Yes | Yes | Yes | Uncertain | Yes | Yes | Yes | Yes | — | — | 87.5 | Low |
| Cushing, 1917([26](#_ENREF_26)) | Yes | Yes | Yes | No | Uncertain | No | Yes | Yes | Yes | NA | 66.7 | Mod |
| Ekpene et al., 2025([55](#_ENREF_55)) | Yes | Yes | Yes | Yes | Yes | Yes | Yes | Yes | — | — | 100 | Low |
| Fischer et al., 2012([39](#_ENREF_39)) | Yes | Yes | Yes | Yes | Yes | Yes | Yes | Yes | — | — | 100 | Low |
| Fujiyama et al., 2024([52](#_ENREF_52)) | Yes | Yes | Yes | Yes | Yes | Yes | Yes | Yes | — | — | 100 | Low |
| Guppy & Ochi, 2018([44](#_ENREF_44)) | Yes | Yes | Yes | Yes | Yes | Yes | Yes | Yes | — | — | 100 | Low |
| Haßler, 1979 ([56](#_ENREF_56)) | No | Yes | Yes | Yes | Uncertain | No | Yes | Yes | Yes | NA | 66.7 | Mod |
| Hoffmann et al., 2014([41](#_ENREF_41)) | Yes | Yes | Yes | Yes | Yes | Yes | Yes | Yes | — | — | 100 | Low |
| Holmes & Sargent, 1915([25](#_ENREF_25)) | Yes | Yes | Yes | Uncertain | Uncertain | Yes | Yes | Yes | — | — | 75 | Low |
| Hopkins, 1885([22](#_ENREF_22)) | Yes | Yes | Yes | NA | Yes | Yes | Yes | Yes | — | — | 100 | Low |
| Horrax, 1919([27](#_ENREF_27)) | Yes | Yes | Yes | Yes | Uncertain | No | Yes | Yes | Yes | NA | 77.8 | Low |
| Judd & Wyatt, 2007([35](#_ENREF_35)) | Yes | Yes | Yes | Uncertain | Yes | Yes | N/A | Yes | — | — | 75 | Low |
| Kapp et al., 1971([28](#_ENREF_28)) | Yes | Yes | Yes | Yes | Yes | Yes | No | Yes | — | — | 87.5 | Low |
| Keen, 1896([24](#_ENREF_24)) | No | Yes | Yes | Uncertain | Yes | Yes | Yes | Yes | — | — | 75 | Low |
| Khursheed et al., 2013([40](#_ENREF_40)) | Yes | Yes | Yes | Yes | Yes | Yes | Yes | Yes | — | — | 100 | Low |
| Kim et al., 2015([42](#_ENREF_42)) | Yes | Yes | Yes | Yes | Yes | Yes | Yes | Yes | — | — | 100 | Low |
| Kow et al., 2023([50](#_ENREF_50)) | Yes | Yes | Yes | Yes | Yes | Yes | Yes | Yes | — | — | 100 | Low |
| Mathew & Sharma, 2010([37](#_ENREF_37)) | Yes | Yes | Yes | Yes | Yes | Yes | Yes | Yes | — | — | 100 | Low |
| Nagahiro et al., 1981([32](#_ENREF_32)) | Yes | Yes | Yes | Yes | Yes | Yes | Yes | No | — | — | 87.5 | Low |
| Nehme, 1980([31](#_ENREF_31)) | Yes | Yes | Yes | Yes | Yes | Yes | Yes | Yes | — | — | 100 | Low |
| Nussbaum et al., 2023([18](#_ENREF_18)) | Yes | Yes | Yes | Yes | Yes | Yes | Yes | Yes | — | — | 100 | Low |
| Olumide & Adeloye, 1976([30](#_ENREF_30)) | Yes | Yes | Yes | Yes | Yes | Yes | Yes | Yes | — | — | 100 | Low |
| Ramos et al., 2017([43](#_ENREF_43)) | Yes | Yes | Yes | Yes | Yes | Yes | Yes | Yes | — | — | 100 | Low |
| Rawdon, 1893([23](#_ENREF_23)) | Yes | Yes | Yes | No | Yes | Yes | Yes | Yes | — | — | 87.5 | Low |
| Sani et al., 2005([34](#_ENREF_34)) | Yes | Yes | Yes | Yes | Yes | Yes | Yes | Yes | — | — | 100 | Low |
| Schlag et al., 2022([49](#_ENREF_49)) | Yes | Yes | Yes | Yes | Yes | Yes | Yes | Yes | — | — | 100 | Low |
| Sedney et al., 2012([38](#_ENREF_38)) | Yes | Yes | Yes | Yes | Yes | Yes | Yes | Yes | — | — | 100 | Low |
| Sheng et al., 2017([6](#_ENREF_6)) | Yes | Yes | Yes | Yes | Yes | Yes | Yes | Yes | — | — | 100 | Low |
| Somrani et al., 2023([51](#_ENREF_51)) | Yes | Yes | Yes | Yes | Yes | Yes | Yes | Yes | — | — | 100 | Low |
| Toogood, 1846([16](#_ENREF_16)) | Yes | Yes | Yes | No | Yes | Yes | Yes | No | — | — | 75 | Low |
| Wu & Shih, 1982([33](#_ENREF_33)) | No | Yes | Yes | No | No | Yes | Yes | Yes | Yes | NA | 66.7 | Mod |
| Zhu et al., 2025([53](#_ENREF_53)) | Yes | Yes | Yes | Yes | Yes | Yes | Yes | Yes | — | — | 100 | Low |
| Zima et al., 2022([47](#_ENREF_47)) | Yes | Yes | Yes | Yes | Yes | Yes | Uncertain | Yes | — | — | 87.5 | Low |

Q1–Q10 refer to questions 1 through 10 from the Joanna Briggs Institute critical appraisal checklist, which applies to either case reports or series, depending on the specific study. Risk of bias was rated as low if 70% or more of the checklist items were met, moderate if 50%–69% were met, and high if less than 50% were met.

Abbreviations: Mod, moderate; NA, not applicable.

**Supplemental Table 2.** American Association of Neurological Surgeons/Congress of Neurological Surgeons (AANS/CNS) classification of evidence.([59](#_ENREF_59))

| **Class** | **Evidence** | **Recommendations** |
| --- | --- | --- |
| I | Rigorously designed randomized controlled trials or systematic reviews/meta-analyses of such trials. | Interventions supported can be strongly recommended with a high degree of certainty in outcomes. |
| II | Well-designed comparative studies such as nonrandomized cohorts, case–control studies. | Interventions supported can be recommended with a moderate degree of certainty, but not definitive evidence. |
| III | Descriptive clinical experience, including case series, case reports, expert consensus. | Interventions may be recommended based on accumulated clinical experience. |
